# A Bicyclic Analog of the Linear Peptide Arodyn Is a Potent and Selective Kappa Opioid Receptor Antagonist

**DOI:** 10.3390/molecules29133109

**Published:** 2024-06-29

**Authors:** Solomon A. Gisemba, Michael J. Ferracane, Thomas F. Murray, Jane V. Aldrich

**Affiliations:** 1 Department of Medicinal Chemistry, University of Florida, Gainesville, FL 32610, USA; contact.sgphd@gmail.com; 2 Department of Chemistry, University of Redlands, Redlands, CA 92373, USA; michael_ferracane@redlands.edu; 3 Department of Pharmacology and Neuroscience, School of Medicine, Creighton University, Omaha, NE 68102, USA; tfmurray@creighton.edu

**Keywords:** kappa opioid receptor (KOR) antagonist, ring-closing metathesis (RCM), tyrosine(O-allyl), dynorphin analog, cyclic peptide, bicyclic peptide

## Abstract

Kappa opioid receptor (KOR) antagonists have potential therapeutic applications in the treatment of stress-induced relapse to substance abuse and mood disorders. The dynorphin A analog arodyn (Ac[Phe^1,2,3^,Arg^4^,D-Ala^8^]dynorphin A-(1–11)-NH_2_) exhibits potent and selective kappa opioid receptor antagonism. Multiple cyclizations in longer peptides, such as dynorphin and its analogs, can extend the conformational constraint to additional regions of the peptide beyond what is typically constrained by a single cyclization. Here, we report the design, synthesis, and pharmacological evaluation of a bicyclic arodyn analog with two constraints in the opioid peptide sequence. The peptide, designed based on structure–activity relationships of monocyclic arodyn analogs, was synthesized by solid-phase peptide synthesis and cyclized by sequential ring-closing metathesis (RCM) in the C- and N-terminal sequences. Molecular modeling studies suggest similar interactions of key aromatic and basic residues in the bicyclic peptide with KOR as found in the cryoEM structure of KOR-bound dynorphin, despite substantial differences in the backbone conformations of the two peptides. The bicyclic peptide’s affinities at KOR and mu opioid receptors (MOR) were determined in radioligand binding assays, and its KOR antagonism was determined in the [^35^S]GTPγS assay in KOR-expressing cells. The bicyclic analog retains KOR affinity and selectivity (K_i_ = 26 nM, 97-fold selectivity over MOR) similar to arodyn and exhibits potent KOR antagonism in the dynorphin-stimulated [^35^S]GTPγS assay. This bicyclic peptide represents a promising advance in preparing cyclic opioid peptide ligands and opens avenues for the rational design of additional bicyclic opioid peptide analogs.

## 1. Introduction

Recently, antagonists of the kappa opioid receptor (KOR) have attracted significant attention due to their potential use in treating various central nervous system (CNS) disorders, especially mood disorders and substance abuse [1,2,3,4,5]. Initial selective antagonists for the KOR, such as JDTic, exhibited prolonged effects, with their action extending from days to weeks following a single injection, which poses challenges for potential clinical advancement (see [4]). Consequently, there has been a growing interest in KOR antagonists, including peptide KOR antagonists [4], with more finite durations of action.

**Figure 1:**
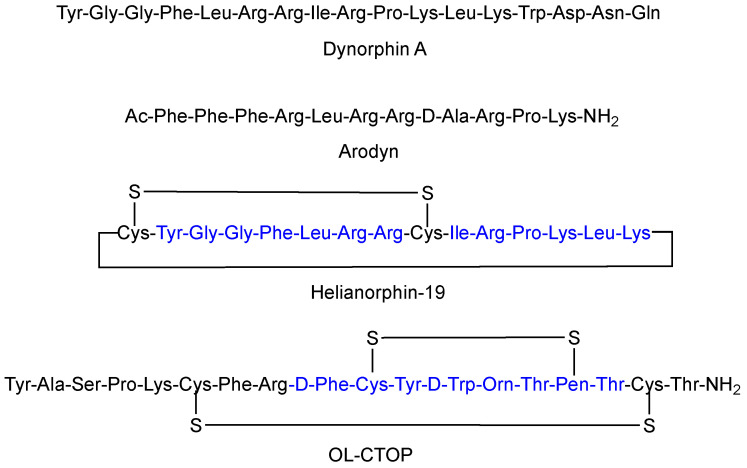
Structures of dynorphin A, arodyn, helianorphin-19 and OLCTOP; the opioid peptide sequences in helianorphin-19 and OL-CTOP are in blue.

The acetylated dynorphin A analog arodyn (Figure 1) is a potent KOR antagonist in vitro and exhibits 174-fold selectivity for KOR over mu opioid receptors (MOR) [6]. In vivo arodyn antagonized antinociception produced by the KOR-selective agonist U50,488 in the mouse 55 °C warm-water tail withdrawal antinociception assay and prevented the reinstatement of cocaine-seeking behavior induced by stress in a mouse conditioned place preference assay [7]. However, the linear structure of this peptide renders it susceptible to rapid breakdown by proteases, thereby restricting its in vivo assessment to central (intracerebroventricular, i.c.v.) administration. 

Cyclizing peptides limits their conformational flexibility, which can stabilize bioactive conformations and confer resistance to proteolysis. As a result, cyclic peptides are highly sought after for their potential to enhance affinity and/or selectivity in comparison to their linear counterparts [8,9,10] while also exhibiting increased metabolic stability [11]. For shorter peptides, including the most extensively researched opioid peptides (enkephalins, deltorphins, etc.), a single cyclization can constrain the majority of the peptide sequence but typically only constrains a portion of the sequence of longer peptides. For opioid peptides, these cyclizations have typically involved disulfide or lactam formation [12,13], although there are some examples of opioid peptide analogs (primarily enkephalin analogs) cyclized by other approaches, including ring-closing metathesis (RCM) [14,15,16,17,18,19].

Consequently, this has spurred the exploration of various techniques to incorporate multiple cyclic constraints into longer peptides to increase conformational constraint and potentially enhance selectivity, potency, and metabolic stability [20]. A variety of cyclization strategies have been employed [21], facilitating the identification of analogs with the desired activity and enhancing structural diversity. 

Examples of multicyclic opioid peptides to date have been limited to two reports where opioid peptides were grafted into non-opioid cyclic peptides. Dynorphin A fragments were grafted into the macrocyclic and bicyclic peptide sunflower trypsin inhibitor-1 (SFTI-1) to stabilize the opioid peptide to degradation, replacing either one of the loops with the dynorphin fragment or splitting dynorphin A-(1–13) across both loops. Incorporating dynorphin A-(1–7) (which contains the basic residues Arg^6^ and Arg^7^ that are important for KOR interaction [22]) into one loop with the remainder of the C-terminal sequence incorporated into the second loop yielded the KOR-selective agonist helianorphin-19 (Figure 1) that retained reasonable KOR affinity (K_i_ = 21 nM vs. 0.3 nM for dynorphin A-(1–13)-NH_2_) and KOR agonist potency (EC_50_ = 43 nM vs. 2.8 nM for dynorphin A-(1–13)-NH_2_) [23]. Recently, the bicyclic peptide OL-CTOP (Figure 1),was reported in which the cyclic MOR antagonist CTOP (D-Phe-*cyclo*[Cys-Tyr-D-Trp-Orn-Thr-Pen]-Thr-NH_2_, Pen = penicillamine) was incorporated into the cyclic peptide odorranelctin (Tyr-Ala-Ser-Pro-Lys-*cyclo*[Cys-Phe-Arg-Tyr-Pro-Asn-Gly-Val-Leu-Ala-Cys]Thr) to facilitate intranasal delivery of CTOP into the brain [24]. To our knowledge, opioid peptides containing multiple cyclizations within the portion of the opioid peptide sequence necessary for its primary receptor interaction have not been reported.

**Figure 2:**
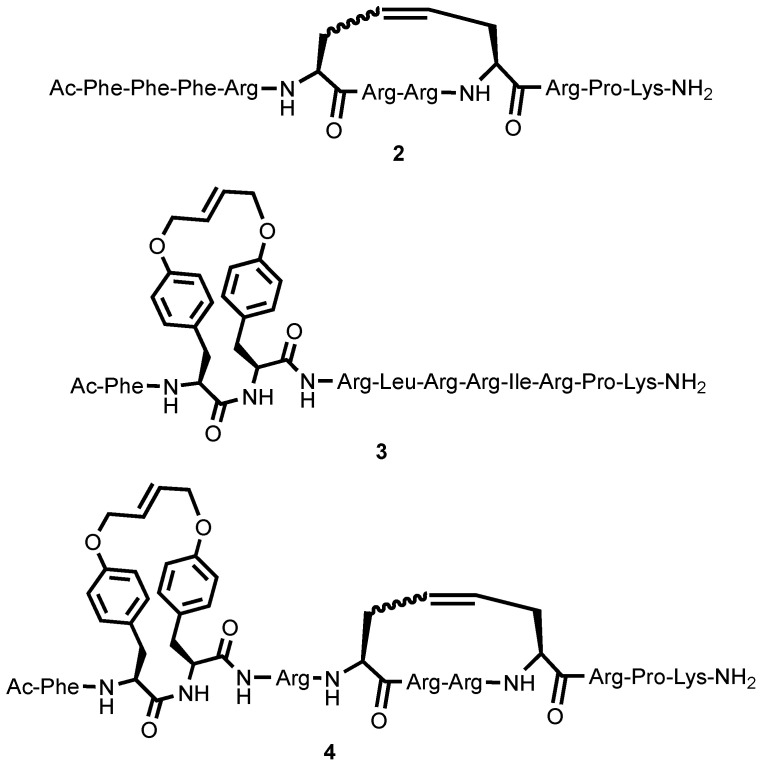
Structures of monocyclic arodyn analogs **2** and **3** and bicyclic analog **4**.

The longer length of dynorphin A and its analogs such as arodyn allows the incorporation of more than one cyclization, extending the portion of the peptide that is constrained. In the initial bicyclic derivative of arodyn reported here, we utilized RCM for the two cyclizations based on the structure–activity relationships (SAR) we developed for arodyn analogs cyclized in different regions of the peptide [25,26,27]. The analog cyclized between allylglycine (AllGly) residues in positions 5 and 8 (**2**) retained KOR affinity, selectivity, and antagonist activity (Table 1), while cyclizations between AllGly in position 2 and position 5 or 8 of arodyn substantially decreased affinity and selectivity for KOR [25]. In contrast, cyclization between Tyr(All) residues in positions 2 and 3 was compatible with KOR interaction (Table 1) [26,27]. Therefore, RCM cyclizations between positions 2 and 3 and between positions 5 and 8 were selected for incorporation into bicyclic analog **4** (Figure 2).

## 2. Results

### 2.1. Synthesis

**Scheme 1:**
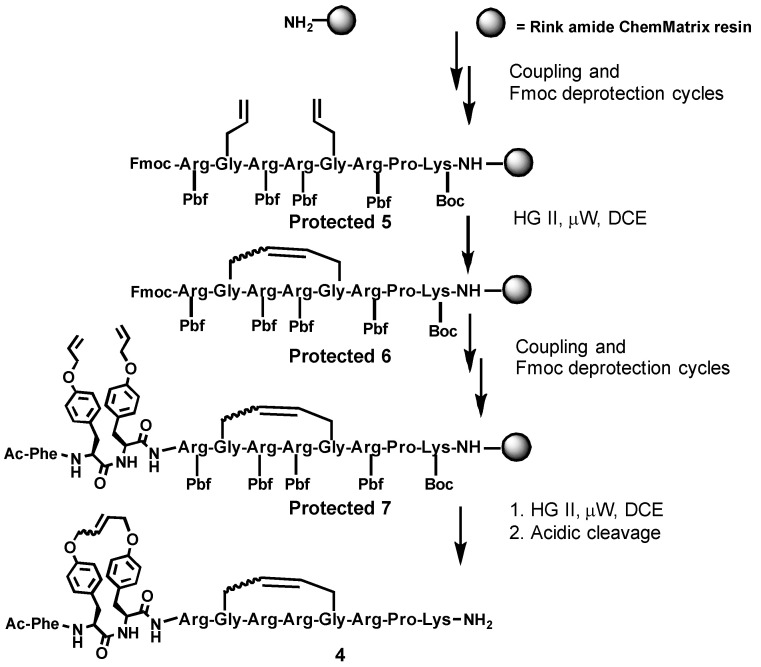
Synthesis of **4** using sequential RCM reactions. DCE, dichloroethane; Pbf, 2,2,4,6,7-pentamethyldihydrobenzofuran-5-sulfonyl.

**Figure 3:**
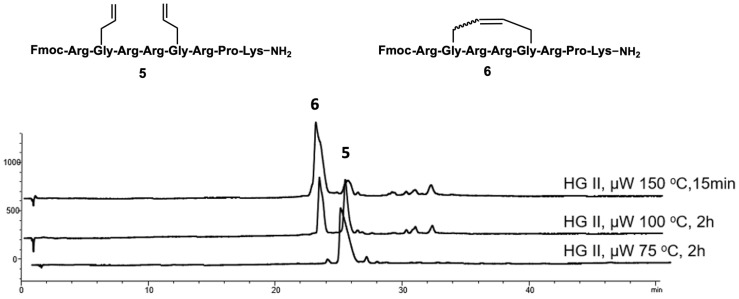
Representative chromatograms showing a temperature-dependent increase in cyclic product yield in the synthesis of the cyclic C-terminal segment to yield peptide **6**. Microwave heating was used in the RCM reaction between the allylglycine residues at positions 5 and 8 at various indicated temperatures.

To obtain regioselectivity, RCM was performed sequentially [28] in the synthesis of bicyclic arodyn analog **4** (Scheme 1), with the RCM performed on the C-terminal segment to yield protected peptide **6** prior to RCM involving the aromatic residues in the N-terminal segment. A temperature-dependent increase in cyclic product yield was observed in the cyclization of the C-terminal segment to give protected peptide **6** (Figure 3), consistent with the temperature-dependent activity of Grubbs catalysts [29,30]. Product yield peaked at 150 °C (63–73%) with no improvement at higher temperatures (see Table S1 in the Supporting Information). The temperature-dependent activity of Grubbs catalysts was also reported by Arora and coworkers, where increasing RCM cyclic peptide yield was observed up to 200 °C with Hoveyda–Grubbs II (HG II) as the catalyst [30].

**Figure 4:**
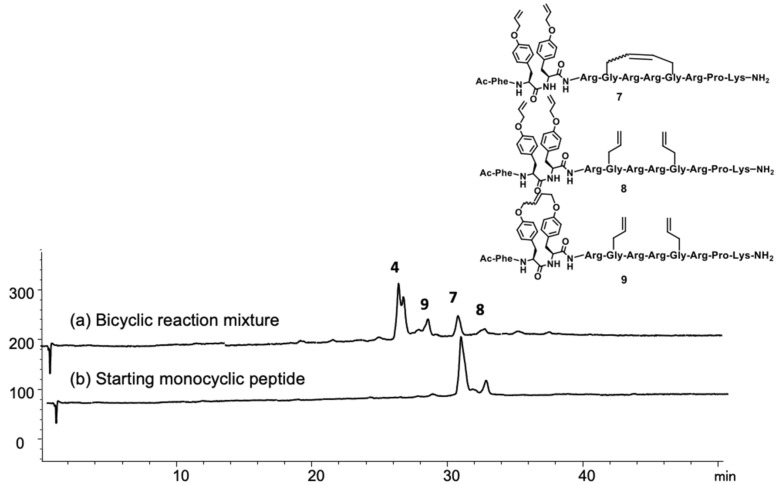
(**a**) Chromatogram of the bicyclic reaction mixture after the second RCM reaction resulting in the bicyclic arodyn analog **4** showing the major components; and (**b**) chromatogram of the monocyclic reaction mixture before the second RCM reaction involving the Tyr(All) residues. For peptide retention times, see Table S2 in the Supporting Information.

Continued solid-phase peptide synthesis (SPPS) resulted in a mixture of the resin-bound protected forms of peptides **7** and **8** (Figure 4). The second RCM was then performed to install the N-terminal heteroatom-containing RCM bridge (Scheme 1). Optimized conditions, previously established to install the heteroatom-containing RCM bridge involving Tyr(All) [31], were used in this step, resulting in the desired bicyclic RCM product **4** in a reasonable yield (52%, Figure 4). The resulting product mixture also contained **7**, **8**, and **9** (Figure 4), as determined by LC-MS analysis. Peptide **9**, the cyclization product of **8**, appeared to be the major side product, consistent with the minimal RCM of AllGly residues at 75 °C for 2 h that was previously observed (Figure 3). 

Purified bicyclic analog **4** was obtained after reversed-phase HPLC purification of the reaction mixture. Side products **7**–**9**, resulting from incomplete RCM reactions, were readily separated from the desired bicyclic peptide **4** during purification. However, the mixture of the *cis* and *trans* isomers of **4** could not be separated. RCM between AllGly residues in positions 5 and 8 yielded both the cis and trans isomers of **2** in approximately equal amounts [25], whereas cyclization between para-substituted Tyr(OAll) residues resulted in exclusively the trans isomer of **3** [26]. Thus, the mixture of isomers in bicyclic peptide **4** is the result of the first RCM between residues 5 and 8.

### 2.2. Molecular Modeling

**Figure 5:**
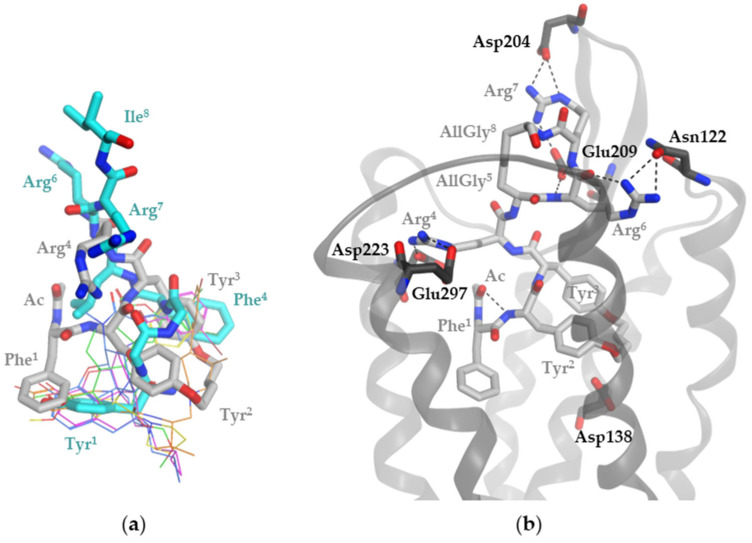
KOR ligands from experimental and model structures: (**a**) overlay of the prior model of the arodyn analog **3** fragment (gray) with experimental structures of dynorphin A-(1–13) (cyan), MP1104 (magenta), JDTic (orange), GR89,696 (yellow), methoxymethyl-salvinorin B (green), and nalfurafine (blue); (**b**) structure of bicyclic arodyn analog **4** (light gray) when docked into the KOR (dark gray), highlighting interactions (black dashes) with important KOR residues.

In a prior study [27], we used molecular modeling to explore how arodyn and its cyclic analogs, including a fragment of analog **3**, might interact with the KOR. Using the crystal structure of JDTic bound to KOR [32] and other opioid receptor structures available at the time, we developed a pharmacophore comprised of two aromatic elements and two positively charged elements to help guide initial placement and subsequent docking of ligand fragments into a KOR model. Additional experimental structures, including that of KOR bound to dynorphin A-(1–13) [22], have been reported [33,34,35,36] that largely support our proposed orientation of residues 1–3 in arodyn and its cyclic analogs in the KOR (Figure 5a).

As such, we again turned to modeling to explore the potential binding of analog **4** and to help rationalize broader SAR observed across arodyn analogs. Briefly, an eight-residue fragment of analog **4** was generated by grafting additional residues onto our analog **3** model fragment; the new residues were allowed to freely rotate to generate a library of conformations that were subsequently docked into a model receptor generated from the structure of KOR bound to dynorphin A-(1–13) [22]. A top pose was chosen that oriented the important groups of the opioid “message” and “address” into appropriate regions of the pocket (Figure 5b).

Consistent with our prior study, the aromatic groups of the analog **4** fragment model (positions 1 and 3) overlay well with those of dynorphin A-(1–13) (positions 1 and 4) and other opioid ligands. Similar to what is observed with methoxymethyl-salvinorin B and KOR [35], no contact is formed between the analog **4** fragment model and the conserved residue Asp138^3.32^; like salvinorin, arodyn and its analogs lack the basic amine present in canonical opioids. These combined findings are consistent with the unique SAR observed for the “message” segment of arodyn and its analogs [37].

The peptide backbone of the analog **4** fragment model differs from that of dynorphin A-(1–13). Similar deviations are seen in other opioid peptides not containing the canonical YGGF “message” and should be expected given the extended, β-strand-like structure of the “address” sequence in dynorphin A-(1–13) and the cyclic, turn-like constraint in the corresponding region of the analog **4** fragment. Still, the backbone falls within favorable/allowable regions of the Ramachandran plot and organizes the carbonyl of position 8 into related space and orientation similar to that of Ile^8^ in dynorphin A-(1–13), the last resolved residue in the experimental structure [22]. Notably, the conformation of the cyclic moiety closely resembles that of related peptide macrocycles [38,39], with the amide groups of the backbone forming contacts with the important, semi-conserved residue Glu209^ECL2^ in the KOR.

In the experimental structure [22], the side chains of Arg^6^ and Arg^7^ in dynorphin A-(1–13) form contacts with Glu209^ECL2^ and Glu297^6.58^ of KOR, respectively. In our modeled fragment-KOR structure, the side chains of Arg^4^, Arg^6^, and Arg^7^ form contacts with Asp223^5.35^/Glu297^6.58^, Asn122^ECL1^, and Asp204^ECL2^/Glu209^ECL2^, respectively; only Asp223^5.35^ is fully conserved across the three opioid receptors. 

As our modeled complex was generated using the active KOR from the dynorphin A-(1–13)-bound experimental structure [22], we envision that the actual complex likely differs due to conformational differences in the receptor (as arodyn and its analogs are antagonists) and induced-fit changes that are not represented here. Still, the combined findings suggest that an energetically accessible conformation of analog **4** can properly orient the important aromatic elements of the “message” and form ionic interactions unique to the “address” of KOR ligands.

### 2.3. Pharmacological Evaluation

**Figure 6:**
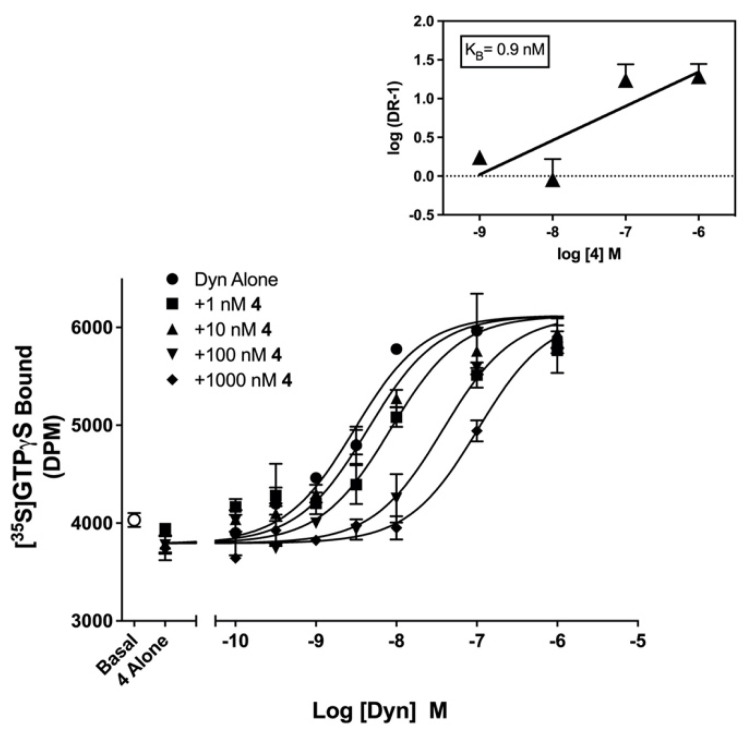
Schild analysis of the antagonism of dynorphin A-(1–13)-NH_2_ (Dyn) by bicyclic arodyn analog **4** at KOR in the [^35^S]GTPγS assay. The Schild plot represents mean values from 4 independent experiments.

The bicyclic peptide **4** was evaluated for its affinity for KOR and MOR in radioligand binding assays and for KOR antagonism in the dynorphin-stimulated [^35^S]GTPγS assay. The bicyclic analog retained affinity at the KOR (K_i_ = 26.4 nM), with a K_i_ value two-fold lower than those for the corresponding monocyclic derivatives **2** and **3** (Table 1). The bicyclic peptide exhibited micromolar affinity for MOR (K_i_ = 2.57 µM), similar to arodyn (Table 1). The 97-fold KOR selectivity over MOR of bicyclic arodyn analog **4** was six-fold higher than the N-terminal cyclized peptide **3** but about two-fold lower than arodyn or the C-terminally cyclized peptide **2**. Like arodyn, in the dynorphin-stimulated [^35^S]GTPγS binding assay, the bicyclic peptide did not exhibit appreciable agonist activity and functioned as a potent competitive KOR antagonist (Figure 6) with low nanomolar potency (K_B_ = 0.9 nM) comparable to that of **3** (K_B_ = 3.2 nM) and **2** (K_B_ = 1.6 and 15 nM for the cis and trans isomers, respectively) [25,26].

## 3. Discussion

Multicyclic peptides are a growing class of macrocycles distinguished by their unique attributes that can be leveraged to modulate biological targets. Notably, multicyclic peptides present considerable promise for therapeutic use and as chemical probes that can exhibit enhanced resistance to proteolytic degradation and improved pharmacokinetic properties [20,40].

In this initial investigation, we designed and synthesized a bicyclic opioid peptide analog of the dynorphin A-based KOR antagonist arodyn (Figure 2). As noted above, previously reported multicyclic opioid peptides have involved grafting an opioid peptide into a non-opioid cyclic peptide to improve metabolic stability or to facilitate delivery [23,24]. To our knowledge, this is the first bicyclic opioid peptide reported in which two cyclic constraints are in the opioid peptide sequence. Leveraging the SAR data for monocyclic arodyn analogs, we selected cyclizations that the KOR tolerated while preserving KOR selectivity. We employed sequential RCM for regioselective cyclization to synthesize the RCM bicyclic analog. Additionally, microwave heating was applied to facilitate the cyclizations, resulting in the successful synthesis of bicyclic arodyn analog **4** in a reasonable yield. Three side products resulted because the RCM reactions did not go to completion, but these were resolved from the bicyclic peptide by HPLC, and **4** could be readily purified. While the RCM cyclization between the two Tyr(All) residues yielded a single isomer [26], cyclizations involving AllGly residues routinely yield mixtures of cis and trans isomers [14,15,16,17,18,19,25]. In some cases, as with **4**, these isomers are unable to be separated. The impact of the double bond configuration on biological activity depends on the positions in the peptide involved in the cyclic constraint and, in some cases, the stereochemistry of the residues involved [18,25]. In the case of the arodyn analog **2,** the cis and trans isomers have similar KOR affinity and selectivity [25], suggesting that the double-bond geometry of the cyclic constraint between residues 5 and 8 does not have a large impact on KOR interaction. 

Molecular modeling suggested that despite substantial differences in the backbone conformation of bicyclic arodyn analog **4** compared to that of dynorphin A-(1–13) bound to KOR [22], key aromatic and basic residues of **4** overlay well with residues in dynorphin A-(1–13) and, in the case of the basic residues, appear to interact with the same acidic residues in KOR. Consistent with our design, bicyclic arodyn analog **4** displayed KOR affinity and KOR antagonism in the dynorphin-stimulated [^35^S]GTPγS assay comparable to monocyclic analogs. 

This bicyclic peptide represents a promising advance in preparing cyclic opioid peptide ligands. Its KOR affinity, selectivity, and antagonist potency demonstrate the potential of multicyclic opioid peptides and open avenues for the rational design of additional bicyclic opioid peptide analogs. However, the SAR of this bicyclic peptide remains to be explored to assess how further structural modification may affect KOR interaction. There is considerable flexibility in the design of additional analogs, including in the positions and identities of the residues involved in the cyclic constraints [27] and the type of linkages that can be utilized in the cyclizations [21], that can be explored in the future. Assessing the pharmacokinetic properties of the peptide will also provide important information to guide additional structural modification. 

## 4. Materials and Methods

### 4.1. Materials

Rink amide ChemMatrix resin was purchased from Biotage (Charlotte, NC, USA). All standard protected amino acids were purchased from Bachem (King of Prussia, PA, USA), EMD Millipore Chemicals (San Diego, CA, USA), Peptides International (Louisville, KY, USA), or Chem-Impex International. All chiral amino acids are of the L-isomer unless otherwise indicated, and abbreviations are used according to the IUPAC-IUB Joint 704 Commission of Biochemical Nomenclature [41]. Fmoc-Tyr(All)-OH and Fmoc-AllGly-OH were purchased from Chem-Impex International (Wood Dale, IL, USA). The coupling agent benzotriazol-1-yl-oxy-tris-pyrrolidino-phosphonium hexafluorophosphate (PyBOP) was obtained from P3 BioSystems (Louisville, KY, USA), and the coupling additive 1-hydroxybenzotriazole hydrate (HOBt) was obtained from Chem-Impex International. Solvents, *N*,*N*-diisopropylethylamine (DIEA), and trifluoroacetic acid (TFA) were purchased from Fisher Scientific (Hampton, NH, USA). All other chemicals were purchased from Aldrich Chemical Co. (Milwaukee, WI, USA).

### 4.2. Instruments

Microwave-assisted RCM and SPPS were performed on a Biotage (Uppsala, Sweden) Initiator+ SP Wave microwave synthesizer and a Biotage microwave peptide synthesizer (Initiator+ Alstra), respectively. 

Analytical HPLC was performed using an Agilent 1200 HPLC system on a Grace Vydac analytical column (C18, 300 Å, 5 μm, 4.6 mm × 50 mm) equipped with a Vydac C18 guard cartridge. Yields for reactions were determined from analytical HPLC chromatograms monitored at 214 nm; baseline resolution was not always achieved, so some yields are estimates. Preparative HPLC was performed using a Shimadzu liquid chromatograph system (with a CBM-20A system controller, LC-20AR solvent delivery module and SPD-20A UV-Vis detector) on a Vydac C18 column (10 μ, 300 Å, 22 × 250 mm) fitted with a C18 guard cartridge; purification was monitored at 214 nm.

Electrospray ionization mass spectrometry (ESI/MS) was performed on an Advion expression L compact mass spectrometer (Advion, Inc., Ithaca, NY, USA).

### 4.3. Synthesis

#### 4.3.1. Solid-Phase Peptide Synthesis 

The Rink amide ChemMatrix resin (0.14 mmol, 0.52 mmol/g) was swelled for 20 min at 70 °C in *N*,*N*-dimethylformamide (DMF) prior to starting the synthesis. Coupling reactions were performed using Fmoc amino acids (4 equiv, 0.5 M), activated with PyBOP (4 equiv, 0.5 M) and HOBt (4 equiv, 0.5 M) in DMF in the presence of DIEA (8 equiv in *N*-methylpyrrolidone, NMP) for 5 min at 75 °C using the Biotage microwave peptide Initiator+ Alstra peptide synthesizer. Fmoc deprotection was performed at room temperature with 20% 4-methylpiperidine in DMF (4.5 mL, 1 × 3 min and 2 × 10 min). The side chains of Lys and Arg were protected with Boc and Pbf, respectively. Following completion of peptide assembly N-terminal acetylation was performed using acetic anhydride (5 M in DMF) and DIEA (2 M in NMP) for 10 min at room temperature. The resin was then washed with DMF (5 mL, 3 × 45 s) and dichloromethane (DCM, 5 mL, 3 × 45 s); the crude peptide was cleaved from the resin using trifluoroacetic acid (TFA)/triisopropylsilane/water (95/2.5/2.5) for at least 2 h and then precipitated with cold ether, followed by filtration.

#### 4.3.2. Ring-Closing Metathesis 

A solution of degassed DCE containing 15 mol % (0.15 equiv) of Hoveyda–Grubbs II catalyst (0.3 mM) was added to the resin-bound peptide (150 mg, 0.21 mmol/g) before heating. Microwave-assisted RCM was performed on the Biotage Initiator+ SP Wave microwave synthesizer in capped microwave reaction vials for the indicated amount of time and temperature; sequential RCM reactions were performed, as shown in Scheme 1. Following washing with MeOH (3 × 5 mL) and DCM (10 × 5 mL) aliquots of resin-bound peptide were cleaved for mass spectral and chromatographic analysis of the peptide product (see Table S2 in the Supporting Information for analytical data for the peptide intermediates).

#### 4.3.3. Synthesis and Purification of Bicyclic Arodyn Analog **4**

Following SPPS of Fmoc-[AllGly^5,8^]arodyn (4–11) (**5**) the protected resin-bound peptide (150 mg, 0.03 mmol, 0.23 mmol/g) was subjected to RCM for 15 min at 150 °C as described above. SPPS was then performed to add the three N-terminal residues, and the resulting full-length resin-bound peptide was subjected to the RCM between the Tyr(All) residues for 2 h at 75 °C as described above (see Scheme 1).

Following cleavage of the final peptide from the resin as described above, crude peptide **4** was purified by preparative reversed-phase HPLC using a linear gradient of 20–40% aqueous MeCN (containing 0.1% TFA) over 80 min at a flow rate of 20 mL/min. Analytical HPLC was used to verify the purity of the final purified peptide **4**: *t*_R_ = 27.0 (99.9% purity) using a linear gradient of 5–50% solvent B (solvent A = aqueous 0.1% TFA and solvent B = MeCN containing 0.1% TFA) over 45 min, and 13.4 min (99.9% purity) using a linear gradient of 25–70% solvent B (solvent A = aqueous 0.1% TFA and solvent B = MeOH containing 0.1% TFA) over 45 min, with both analyses at a flow rate of 1.0 mL/min; *m*/*z* 801.7 ([M + 2H]^2+^) and 534.9 ([M + 3H]^3+^), calcd. *m*/*z* 801.5 ([M + 2H]^2+^) and 534.6 ([M + 3H]^3+^).

### 4.4. Molecular Modeling

Molecular modeling was performed using the 2022 Molecular Operating Environment (MOE 2022.02) software suite available from the Chemical Computing Group (CCG, Montreal, QC, Canada [42]. Our prior model of cyclic arodyn analog **3** docked in the KOR [27] as well as experimental structures of dynorphin A-(1–13) [22], MP1104 [33], JDTic [32], GR89,696 [35], methoxymethyl-salvinorin B [35], DNCP-β-NalA [36], and nalfurafine [34] with KOR were superimposed and used for structural comparison. 

To generate bicyclic arodyn analog **4**, residues 5–8 were grafted onto our prior model of cyclic arodyn analog **3** and the C-terminus was *N*-methylated to better approximate the steric and electronic environment of the full peptide. Residues 1–3 were held fixed while the rest of the peptide underwent 50,000 iterations of stochastic bond bending, stretching, and rotation using the Amber10:EHT forcefield [43], resulting in 50,000 unique conformers that were saved into a library. 

Holding both the ligand and the receptor rigid, the entire conformer library was docked into a KOR model prepared from the experimental KOR-dynorphin A-(1–13) structure, as done previously [27]. A top preliminary structure was chosen from these results, ensuring that (a) dihedral angles of the peptide backbone fell into acceptable regions of the Ramachandran plot, (b) no cis amide bonds were present outside the cyclic portions of the peptide, (c) residue 8 in the analog **4** fragment and Ile^8^ in dynorphin A-(1–13) had similar orientations of their carbonyls and fell within 2.0 Å of each other, and (d) the arginine side chains of analog **4** were directed toward acidic/polar residues of the KOR or solvent. Following this, the side chains of Arg^4^, Arg^6^, and Arg^7^ in the peptide, as well as Arg202^ECL2^, Asp204^ECL2^, Glu209^ECL2^, Asp223^5.35^, and Glu297^6.58^ in KOR, were allowed to freely move and co-minimized using the Amber10:EHT forcefield. Finally, the entire peptide and all KOR residues containing atoms within 4.5 Å of it were allowed to freely move and co-minimized to yield the final model complex.

### 4.5. Pharmacological Evaluation

The binding affinities of **4** for KOR and MOR were determined by competitive inhibition of [^3^H]diprenorphine and [^3^H]DAMGO ([D-Ala^2^,MePhe^4^, glyol]enkephalin) binding to cloned rat KOR and MOR, respectively, stably expressed on Chinese hamster ovary (CHO) cells following previously reported procedures [27,44]. K_i_ values were calculated from the IC_50_ values with the Cheng and Prusoff equation [45] using K_D_ values of 0.45 and 0.49 for [^3^H]diprenorphine and [^3^H]DAMGO, respectively. The mean K_i_ values ± SEM were calculated from at least 3 independent experiments. Arodyn has low affinity for the ẟ opioid receptor (DOR) (K_i_ > 5 µM) [6]; therefore, the affinities of the analogs for this receptor were not evaluated.

The stimulation of binding of the GTP analog [^35^S]GTPγS to membranes containing KOR was assayed in the presence of peptidase inhibitors, as described previously [25,26,27]. The antagonist potency of **4** at KOR was determined by measuring the EC_50_ values of dynorphin A-(1–13)NH_2_ in the absence and presence of four different concentrations (1–1000 nM) of bicyclic peptide **4**. The pA_2_ value was determined by Schild analysis [46]; the data are reported as the K_B_ value.

## Figures and Tables

**Table 1:** Affinities of arodyn, monocyclic RCM arodyn analogs, and the bicyclic analog **4** for KOR and MOR.

Peptide	KOR K_i_ ± SEM (nM)	MOR K_i_ ± SEM (nM)	K_i_ Ratio (MOR/KOR)
**1**, arodyn ^1^	10.0 ± 3.0	1750 ± 130	174
**2** ^2,3^	54.0 ± 3.9/ 63.0 ± 6.3	>10,000/ 9370 ± 590	>185/ 149
**3** ^4,5^	55.4 ± 4.1	903 ± 34 ^1^	16
**4**	26.4 ± 5.9	2570 ± 500	97

^1^ ref. [6]; ^2^ cis/trans isomers; ^3^ ref. [25]; ^4^ trans isomer; ^5^ ref. [26].

## Data Availability

The raw data supporting the conclusions of this study will be made available by the corresponding author upon request.

## Supplementary Materials

The following supporting information can be downloaded at https://www.mdpi.com/article/10.3390/molecules29133109/s1, Figure S1: HPLC chromatograms of pure bicyclic peptide **4**; Figure S2: Electrospray ionization mass spectrum of pure bicyclic peptide **4**; Table S1: Yield of intermediate **6** following RCM with HG II under various microwave heating conditions; Table S2: Analytical data for peptides **5**–**9**; PDB file of analog **4** docked to KOR.
