# Supplementary material for: A Bicyclic Analog of the Linear Peptide Arodyn Is a Potent and Selective Kappa Opioid Receptor Antagonist
Source: Molecules. 2024 Jun 29;29(13):3109. doi: 10.3390/molecules29133109 (PMC11243530; doi:10.3390/molecules29133109)

Supplementary Materials

A Bicyclic Analog of the Linear Peptide Arodyn Is a Potent and Selective Kappa Opioid Receptor Antagonist

Solomon A. Gisemba [1], Michael J. Ferracane [2], Thomas F. Murray [3] and Jane V. Aldrich [1,*]

1 Department of Medicinal Chemistry, University of Florida, Gainesville, FL 32610, USA; contact.sgphd@gmail.com

2 Department of Chemistry, University of Redlands, Redlands, CA 92373, USA; michael_ferracane@redlands.edu

3 Department of Pharmacology and Neuroscience, School of Medicine, Creighton University, Omaha, NE 68102, USA; tfmurray@creighton.edu

* Correspondence: janealdrich@ufl.edu

Table of Contents

	Page
Table S1. Yield of intermediate **6** following RCM with HG II under various microwave heating conditions.	2
Table S2. Analytical data for peptides **5-9**.	2
Figure S1. HPLC chromatograms of pure bicyclic peptide **4**.	3
Figure S2. Electrospray ionization mass spectrum of pure bicyclic peptide **4**.	3

Table S1. Yield of intermediate **6** following RCM with HG II under various microwave heating conditions.[a,b]

Entry	Temp (°C)	Time	RCM Pdt yield (%)
1	75	2 h	-[c]
2	100	2 h	46
3	150	5 min	63
4	150	15 min	73
5	150	30 min	73
6	175	15 min	67

[a]15 mol% HG II, 0.3 mM, in DCE; [b]product yield was determined by analytical HPLC (5-50% aqueous MeCN containing 0.1% TFA over 45 min; see Table S2 for retention times of products); [c] <10%

Table S2. Analytical data for peptides **5-9**.

Compound	HPLC t_R (min)[a]	ESI-MS	
		Calcd.	Obsvd.
**5**	25.3	$[M+3H]^{3+}$ 428.6	$[M+3H]^{3+}$ 428.7
**6**	23.3	$[M+3H]^{3+}$ 419.2	$[M+3H]^{3+}$ 419.5
**7**	30.8	$[M+3H]^{3+}$ 544.0<br>$[M+4H]^{4+}$ 408.2	$[M+3H]^{3+}$ 543.9<br>$[M+4H]^{4+}$ 408.2
**8**	32.9	$[M+3H]^{3+}$ 553.3<br>$[M+4H]^{4+}$ 415.3	$[M+3H]^{3+}$ 553.2<br>$[M+4H]^{4+}$ 415.2
**9**	28.7	$[M+2H]^{2+}$ 815.5<br>$[M+3H]^{3+}$ 544.0	$[M+2H]^{2+}$ 815.4<br>$[M+3H]^{3+}$ 543.9

[a]5-50% aqueous MeCN (0.1% TFA) over 45 min

Figure S1. HPLC chromatograms of pure bicyclic peptide **4**.

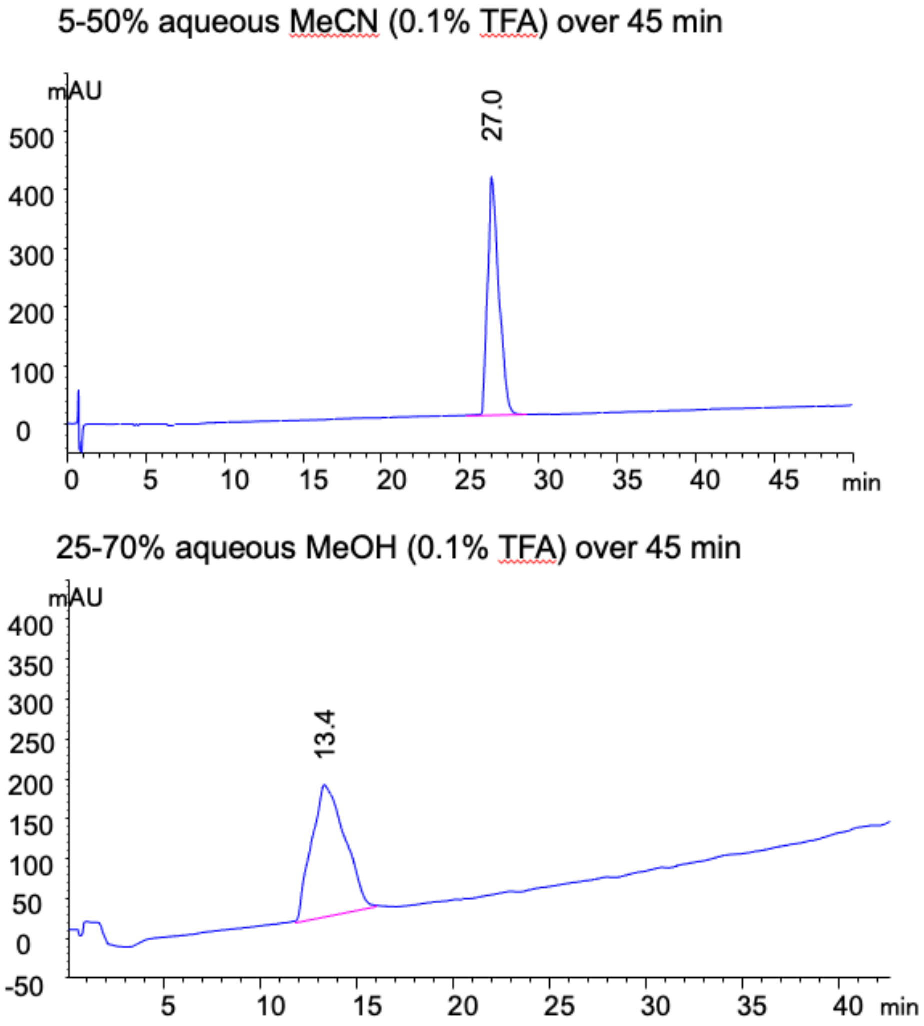

Figure S2. Electrospray ionization mass spectrum of pure bicyclic peptide **4**.

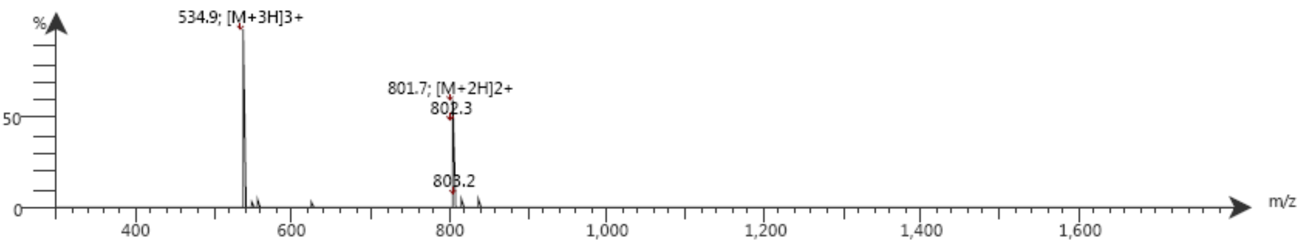

Supplement: Supplementary file 1 [file molecules-29-03109-s001.zip › molecules-2953644-supplementary.pdf]
